# *trans*-Diiodidobis(2-phenyl­pyridine-κ*N*)palladium(II)

**DOI:** 10.1107/S1600536811055425

**Published:** 2012-01-07

**Authors:** Kwang Ha

**Affiliations:** a School of Applied Chemical Engineering, The Research Institute of Catalysis, Chonnam National University, Gwangju 500-757, Republic of Korea

## Abstract

In the title complex, [PdI_2_(C_11_H_9_N)_2_], the Pd^II^ ion has a distorted *trans*-I_2_N_2_ square-planar coordination geometry defined by two N atoms from two 2-phenyl­pyridine ligands and two I^−^ anions. The 2-phenyl­pyridine ligands are not planar, the dihedral angles between the pyridine and benzene rings being 50.1 (2) and 45.7 (2)°. An inter­molecular π–π inter­action between the six-membered rings is present, the ring centroid–centroid distance being 3.898 (4) Å.

## Related literature

For a related structure, [PdCl_2_(C_11_H_9_N)_2_], see: Ha (2011 ▶).
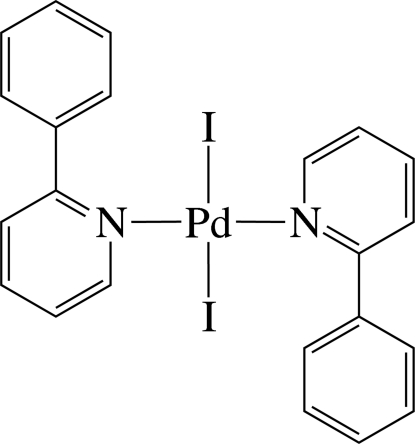


## Experimental

### 

#### Crystal data

[PdI_2_(C_11_H_9_N)_2_]
*M* *_r_* = 670.58
Monoclinic, 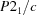

*a* = 9.9163 (10) Å
*b* = 14.4759 (14) Å
*c* = 14.9917 (15) Å
β = 103.663 (2)°
*V* = 2091.1 (4) Å^3^
*Z* = 4
Mo *K*α radiation
μ = 3.85 mm^−1^
*T* = 200 K
0.25 × 0.23 × 0.11 mm

#### Data collection

Bruker SMART 1000 CCD diffractometer
Absorption correction: multi-scan (*SADABS*; Bruker, 2000 ▶) *T*_min_ = 0.511, *T*_max_ = 0.655
15087 measured reflections
5163 independent reflections
2650 reflections with *I* > 2σ(*I*)
*R*_int_ = 0.061

#### Refinement

*R*[*F* ^2^ > 2σ(*F* ^2^)] = 0.041
*wR*(*F* ^2^) = 0.110
*S* = 0.98
5163 reflections
244 parameters
H-atom parameters constrained
Δρ_max_ = 2.20 e Å^−3^
Δρ_min_ = −1.15 e Å^−3^

### 

Data collection: *SMART* (Bruker, 2000 ▶); cell refinement: *SAINT* (Bruker, 2000 ▶); data reduction: *SAINT*; program(s) used to solve structure: *SHELXS97* (Sheldrick, 2008 ▶); program(s) used to refine structure: *SHELXL97* (Sheldrick, 2008 ▶); molecular graphics: *ORTEP-3* (Farrugia, 1997 ▶) and *PLATON* (Spek, 2009 ▶); software used to prepare material for publication: *SHELXL97*.

## Supplementary Material

Crystal structure: contains datablock(s) global, I. DOI: 10.1107/S1600536811055425/is5038sup1.cif

Structure factors: contains datablock(s) I. DOI: 10.1107/S1600536811055425/is5038Isup2.hkl

Additional supplementary materials:  crystallographic information; 3D view; checkCIF report

## Figures and Tables

**Table 1:** Selected bond lengths (Å)

Pd1—N1	2.027 (5)
Pd1—N2	2.031 (5)
Pd1—I1	2.6178 (8)
Pd1—I2	2.6244 (8)

**Fig. 1.**
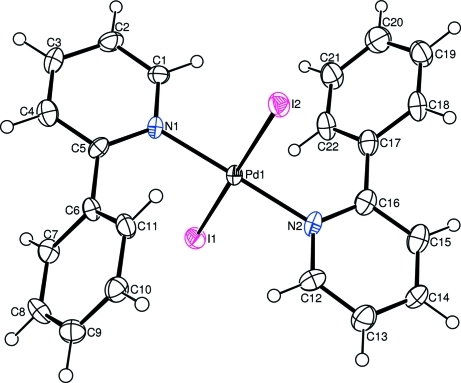
A view of the molecular structure of the title complex, with displacement ellipsoids drawn at the 40% probability level and the atom numbering.

**Fig. 2.**
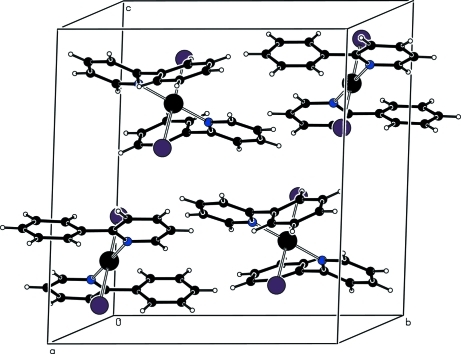
A view of the unit-cell contents of the title complex.

## supplementary crystallographic information

### Comment

The title complex, [PdI_2_(C_11_H_9_N)_2_], crystallized in the monoclinic space group *P*2_1_/*c*, whereas the analogous chloro Pd^II^ complex [PdCl_2_(C_11_H_9_N)_2_] crystallized in the triclinic space group *P*1 (Ha, 2011).

The central Pd^II^ ion has a *trans*-I_2_N_2_ square-planar coordination geometry defined by two N atoms from two 2-phenylpyridine ligands and two I^-^ anions (Fig. 1). The Pd—N and Pd—I bond lengths are nearly equivalent, respectively (Table 1). In the crystal, the PdI_2_N_2_ unit is nearly planar: the maximum deviation from the least-squares plane is 0.002 (2) Å. The dihedral angles between the PdI_2_N_2_ moiety and the pyridine rings are 77.2 (2) and 76.8 (2)°. The 2-phenylpyridine ligands are not planar, the dihedral angles between the pyridine and benzene rings being 50.1 (2)° and 45.7 (2)°. An intermolecular π–π interaction between the six-membered rings is present, the ring centroid-centroid distance being 3.898 (4) Å (Fig. 2).

### Experimental

To a solution of Na_2_PdCl_4_ (0.1494 g, 0.508 mmol) and KI (0.9225 g, 5.557 mmol) in MeOH (50 ml) was added 2-phenylpyridine (0.1828 g, 1.178 mmol) and stirred for 7 h at room temperature. After evaporation of the solvent, the residue was washed with H_2_O and dried at 50 °C, to give a redbrown powder (0.3430 g). Crystals suitable for X-ray analysis were obtained by slow evaporation from a CH_3_CN solution.

### Refinement

H atoms were positioned geometrically and allowed to ride on their respective parent atoms (C—H = 0.95 Å and *U*_iso_(H) = 1.2*U*_eq_(C)). The highest peak (2.20 e Å^-3^) and the deepest hole (-1.15 e Å^-3^) in the difference Fourier map are located 1.00 Å and 0.86 Å from the atoms H10 and Pd1, respectively.

### Crystal data

**Table:**

[PdI_2_(C_11_H_9_N)_2_]	*F*(000) = 1264
*M**_r_* = 670.58	*D*_x_ = 2.130 Mg m^−^^3^
Monoclinic, *P*2_1_/*c*	Mo *K*α radiation, λ = 0.71073 Å
Hall symbol: -P 2ybc	Cell parameters from 3071 reflections
*a* = 9.9163 (10) Å	θ = 2.5–27.7°
*b* = 14.4759 (14) Å	µ = 3.85 mm^−^^1^
*c* = 14.9917 (15) Å	*T* = 200 K
β = 103.663 (2)°	Plate, red
*V* = 2091.1 (4) Å^3^	0.25 × 0.23 × 0.11 mm
*Z* = 4	

### Data collection

**Table:**

Bruker SMART 1000 CCD diffractometer	5163 independent reflections
Radiation source: fine-focus sealed tube	2650 reflections with *I* > 2σ(*I*)
graphite	*R*_int_ = 0.061
φ and ω scans	θ_max_ = 28.3°, θ_min_ = 2.0°
Absorption correction: multi-scan (*SADABS*; Bruker, 2000)	*h* = −12→13
*T*_min_ = 0.511, *T*_max_ = 0.655	*k* = −18→19
15087 measured reflections	*l* = −19→17

### Refinement

**Table:**

Refinement on *F*^2^	Primary atom site location: structure-invariant direct methods
Least-squares matrix: full	Secondary atom site location: difference Fourier map
*R*[*F*^2^ > 2σ(*F*^2^)] = 0.041	Hydrogen site location: inferred from neighbouring sites
*wR*(*F*^2^) = 0.110	H-atom parameters constrained
*S* = 0.98	*w* = 1/[σ^2^(*F*_o_^2^) + (0.0329*P*)^2^] where *P* = (*F*_o_^2^ + 2*F*_c_^2^)/3
5163 reflections	(Δ/σ)_max_ < 0.001
244 parameters	Δρ_max_ = 2.20 e Å^−^^3^
0 restraints	Δρ_min_ = −1.15 e Å^−^^3^

### Special details

**Table:**

Geometry. All e.s.d.'s (except the e.s.d. in the dihedral angle between two l.s. planes) are estimated using the full covariance matrix. The cell e.s.d.'s are taken into account individually in the estimation of e.s.d.'s in distances, angles and torsion angles; correlations between e.s.d.'s in cell parameters are only used when they are defined by crystal symmetry. An approximate (isotropic) treatment of cell e.s.d.'s is used for estimating e.s.d.'s involving l.s. planes.
Refinement. Refinement of *F*^2^ against ALL reflections. The weighted *R*-factor *wR* and goodness of fit *S* are based on *F*^2^, conventional *R*-factors *R* are based on *F*, with *F* set to zero for negative *F*^2^. The threshold expression of *F*^2^ > σ(*F*^2^) is used only for calculating *R*-factors(gt) *etc*. and is not relevant to the choice of reflections for refinement. *R*-factors based on *F*^2^ are statistically about twice as large as those based on *F*, and *R*- factors based on ALL data will be even larger.

### Fractional atomic coordinates and isotropic or equivalent isotropic displacement parameters (Å^2^)

**Table:**

	*x*	*y*	*z*	*U*_iso_*/*U*_eq_	
Pd1	0.75033 (5)	0.14770 (3)	0.24074 (3)	0.02261 (13)	
I1	0.61261 (5)	0.14579 (3)	0.36973 (3)	0.03177 (14)	
I2	0.88936 (5)	0.15018 (3)	0.11195 (3)	0.03134 (14)	
N1	0.6117 (5)	0.0560 (3)	0.1689 (3)	0.0223 (12)	
N2	0.8874 (6)	0.2392 (4)	0.3151 (4)	0.0294 (14)	
C1	0.6530 (7)	−0.0331 (4)	0.1743 (4)	0.0282 (16)	
H1	0.7465	−0.0464	0.2043	0.034*	
C2	0.5691 (8)	−0.1047 (5)	0.1396 (5)	0.0365 (19)	
H2	0.6031	−0.1663	0.1452	0.044*	
C3	0.4332 (8)	−0.0863 (5)	0.0959 (5)	0.0339 (18)	
H3	0.3708	−0.1349	0.0720	0.041*	
C4	0.3903 (7)	0.0041 (5)	0.0877 (5)	0.0325 (18)	
H4	0.2980	0.0181	0.0556	0.039*	
C5	0.4787 (7)	0.0753 (4)	0.1254 (4)	0.0249 (16)	
C6	0.4288 (7)	0.1721 (4)	0.1201 (4)	0.0252 (16)	
C7	0.3044 (7)	0.1915 (5)	0.1446 (4)	0.0281 (16)	
H7	0.2540	0.1430	0.1645	0.034*	
C8	0.2542 (8)	0.2810 (5)	0.1401 (5)	0.0382 (19)	
H8	0.1710	0.2940	0.1587	0.046*	
C9	0.3244 (8)	0.3508 (5)	0.1090 (5)	0.0354 (18)	
H9	0.2890	0.4120	0.1056	0.043*	
C10	0.4459 (7)	0.3331 (5)	0.0824 (5)	0.0319 (18)	
H10	0.4938	0.3821	0.0610	0.038*	
C11	0.4991 (7)	0.2430 (5)	0.0868 (4)	0.0299 (17)	
H11	0.5818	0.2303	0.0674	0.036*	
C12	0.8459 (8)	0.3265 (5)	0.3126 (5)	0.0348 (19)	
H12	0.7536	0.3406	0.2805	0.042*	
C13	0.9289 (8)	0.3975 (5)	0.3540 (5)	0.043 (2)	
H13	0.8956	0.4592	0.3496	0.052*	
C14	1.0588 (8)	0.3775 (5)	0.4010 (5)	0.040 (2)	
H14	1.1177	0.4255	0.4310	0.048*	
C15	1.1072 (8)	0.2880 (5)	0.4060 (5)	0.042 (2)	
H15	1.1990	0.2742	0.4393	0.051*	
C16	1.0189 (8)	0.2168 (5)	0.3612 (5)	0.0321 (18)	
C17	1.0712 (8)	0.1208 (5)	0.3653 (5)	0.0351 (19)	
C18	1.2040 (8)	0.1041 (6)	0.3535 (5)	0.041 (2)	
H18	1.2584	0.1540	0.3402	0.049*	
C19	1.2577 (8)	0.0150 (5)	0.3609 (5)	0.044 (2)	
H19	1.3474	0.0041	0.3508	0.053*	
C20	1.1810 (9)	−0.0581 (6)	0.3831 (5)	0.049 (2)	
H20	1.2183	−0.1189	0.3893	0.059*	
C21	1.0499 (8)	−0.0414 (5)	0.3962 (5)	0.044 (2)	
H21	0.9967	−0.0909	0.4116	0.053*	
C22	0.9956 (8)	0.0471 (5)	0.3871 (5)	0.041 (2)	
H22	0.9050	0.0576	0.3958	0.049*	

### Atomic displacement parameters (Å^2^)

**Table:**

	*U*^11^	*U*^22^	*U*^33^	*U*^12^	*U*^13^	*U*^23^
Pd1	0.0173 (3)	0.0242 (3)	0.0258 (3)	−0.0034 (2)	0.0040 (2)	−0.0018 (2)
I1	0.0291 (3)	0.0354 (3)	0.0328 (3)	−0.0015 (2)	0.0115 (2)	−0.0018 (2)
I2	0.0253 (3)	0.0370 (3)	0.0331 (3)	−0.0029 (2)	0.0095 (2)	−0.0013 (2)
N1	0.017 (3)	0.027 (3)	0.021 (3)	−0.005 (2)	0.001 (2)	0.001 (2)
N2	0.026 (4)	0.031 (3)	0.029 (3)	−0.011 (3)	0.002 (3)	−0.001 (3)
C1	0.029 (4)	0.024 (4)	0.032 (4)	0.002 (3)	0.009 (3)	−0.003 (3)
C2	0.045 (5)	0.021 (4)	0.044 (5)	−0.001 (3)	0.011 (4)	−0.005 (3)
C3	0.030 (5)	0.038 (4)	0.032 (4)	−0.013 (4)	0.003 (3)	−0.002 (3)
C4	0.030 (5)	0.041 (5)	0.024 (4)	0.002 (4)	0.001 (3)	0.009 (3)
C5	0.031 (4)	0.025 (4)	0.016 (3)	−0.004 (3)	0.002 (3)	−0.002 (3)
C6	0.018 (4)	0.030 (4)	0.025 (4)	0.002 (3)	0.001 (3)	0.005 (3)
C7	0.026 (4)	0.036 (4)	0.026 (4)	−0.006 (3)	0.014 (3)	0.001 (3)
C8	0.023 (5)	0.046 (5)	0.046 (5)	0.012 (4)	0.010 (3)	0.003 (4)
C9	0.037 (5)	0.026 (4)	0.042 (4)	0.003 (4)	0.007 (3)	0.004 (4)
C10	0.030 (5)	0.034 (4)	0.034 (4)	−0.005 (3)	0.011 (3)	−0.001 (3)
C11	0.020 (4)	0.038 (4)	0.029 (4)	0.003 (3)	0.002 (3)	0.000 (3)
C12	0.046 (5)	0.024 (4)	0.035 (4)	−0.003 (3)	0.011 (4)	−0.002 (3)
C13	0.044 (6)	0.034 (5)	0.053 (5)	−0.010 (4)	0.014 (4)	−0.002 (4)
C14	0.039 (5)	0.036 (5)	0.043 (5)	−0.010 (4)	0.005 (4)	−0.018 (4)
C15	0.039 (5)	0.050 (5)	0.034 (4)	−0.012 (4)	−0.001 (4)	−0.002 (4)
C16	0.036 (5)	0.033 (4)	0.028 (4)	−0.010 (3)	0.010 (3)	0.006 (3)
C17	0.028 (5)	0.045 (5)	0.028 (4)	−0.003 (4)	0.000 (3)	0.002 (3)
C18	0.023 (5)	0.054 (5)	0.042 (5)	−0.005 (4)	0.000 (4)	0.005 (4)
C19	0.032 (5)	0.046 (5)	0.053 (5)	0.004 (4)	0.008 (4)	0.003 (4)
C20	0.047 (6)	0.047 (5)	0.047 (5)	0.008 (4)	−0.002 (4)	0.001 (4)
C21	0.036 (5)	0.036 (5)	0.059 (5)	−0.001 (4)	0.012 (4)	0.012 (4)
C22	0.024 (5)	0.050 (5)	0.044 (5)	−0.002 (4)	0.001 (3)	0.007 (4)

### Geometric parameters (Å, °)

**Table:**

Pd1—N1	2.027 (5)	C9—H9	0.9500
Pd1—N2	2.031 (5)	C10—C11	1.402 (9)
Pd1—I1	2.6178 (8)	C10—H10	0.9500
Pd1—I2	2.6244 (8)	C11—H11	0.9500
N1—C1	1.350 (8)	C12—C13	1.369 (9)
N1—C5	1.356 (8)	C12—H12	0.9500
N2—C12	1.328 (8)	C13—C14	1.346 (9)
N2—C16	1.363 (8)	C13—H13	0.9500
C1—C2	1.354 (9)	C14—C15	1.378 (10)
C1—H1	0.9500	C14—H14	0.9500
C2—C3	1.378 (9)	C15—C16	1.415 (9)
C2—H2	0.9500	C15—H15	0.9500
C3—C4	1.372 (9)	C16—C17	1.481 (10)
C3—H3	0.9500	C17—C22	1.386 (10)
C4—C5	1.384 (8)	C17—C18	1.390 (10)
C4—H4	0.9500	C18—C19	1.389 (10)
C5—C6	1.482 (9)	C18—H18	0.9500
C6—C11	1.397 (9)	C19—C20	1.389 (10)
C6—C7	1.397 (9)	C19—H19	0.9500
C7—C8	1.384 (9)	C20—C21	1.381 (11)
C7—H7	0.9500	C20—H20	0.9500
C8—C9	1.370 (10)	C21—C22	1.385 (10)
C8—H8	0.9500	C21—H21	0.9500
C9—C10	1.380 (10)	C22—H22	0.9500
			
N1—Pd1—N2	178.8 (2)	C9—C10—C11	120.2 (7)
N1—Pd1—I1	88.78 (15)	C9—C10—H10	119.9
N2—Pd1—I1	89.99 (17)	C11—C10—H10	119.9
N1—Pd1—I2	91.51 (15)	C6—C11—C10	119.1 (7)
N2—Pd1—I2	89.71 (17)	C6—C11—H11	120.5
I1—Pd1—I2	179.71 (3)	C10—C11—H11	120.5
C1—N1—C5	118.2 (5)	N2—C12—C13	123.6 (7)
C1—N1—Pd1	115.8 (4)	N2—C12—H12	118.2
C5—N1—Pd1	125.6 (4)	C13—C12—H12	118.2
C12—N2—C16	119.5 (6)	C14—C13—C12	118.3 (7)
C12—N2—Pd1	116.3 (5)	C14—C13—H13	120.8
C16—N2—Pd1	124.0 (5)	C12—C13—H13	120.8
N1—C1—C2	124.0 (6)	C13—C14—C15	120.6 (7)
N1—C1—H1	118.0	C13—C14—H14	119.7
C2—C1—H1	118.0	C15—C14—H14	119.7
C1—C2—C3	118.5 (7)	C14—C15—C16	119.3 (7)
C1—C2—H2	120.7	C14—C15—H15	120.3
C3—C2—H2	120.7	C16—C15—H15	120.3
C4—C3—C2	118.4 (6)	N2—C16—C15	118.6 (7)
C4—C3—H3	120.8	N2—C16—C17	121.9 (6)
C2—C3—H3	120.8	C15—C16—C17	119.5 (7)
C3—C4—C5	121.4 (6)	C22—C17—C18	118.6 (7)
C3—C4—H4	119.3	C22—C17—C16	121.9 (7)
C5—C4—H4	119.3	C18—C17—C16	119.4 (7)
N1—C5—C4	119.6 (6)	C19—C18—C17	120.5 (8)
N1—C5—C6	119.7 (5)	C19—C18—H18	119.8
C4—C5—C6	120.7 (6)	C17—C18—H18	119.8
C11—C6—C7	119.5 (6)	C20—C19—C18	120.3 (8)
C11—C6—C5	121.7 (7)	C20—C19—H19	119.8
C7—C6—C5	118.7 (6)	C18—C19—H19	119.8
C8—C7—C6	120.3 (7)	C21—C20—C19	119.2 (8)
C8—C7—H7	119.8	C21—C20—H20	120.4
C6—C7—H7	119.8	C19—C20—H20	120.4
C9—C8—C7	120.1 (7)	C20—C21—C22	120.4 (8)
C9—C8—H8	119.9	C20—C21—H21	119.8
C7—C8—H8	119.9	C22—C21—H21	119.8
C8—C9—C10	120.6 (7)	C21—C22—C17	121.0 (8)
C8—C9—H9	119.7	C21—C22—H22	119.5
C10—C9—H9	119.7	C17—C22—H22	119.5
			
I1—Pd1—N1—C1	−100.1 (5)	C8—C9—C10—C11	0.1 (11)
I2—Pd1—N1—C1	79.8 (5)	C7—C6—C11—C10	2.6 (9)
I1—Pd1—N1—C5	72.3 (5)	C5—C6—C11—C10	179.2 (6)
I2—Pd1—N1—C5	−107.7 (5)	C9—C10—C11—C6	−1.2 (10)
I1—Pd1—N2—C12	−78.3 (5)	C16—N2—C12—C13	0.5 (11)
I2—Pd1—N2—C12	101.7 (5)	Pd1—N2—C12—C13	−175.6 (6)
I1—Pd1—N2—C16	105.8 (6)	N2—C12—C13—C14	−1.2 (12)
I2—Pd1—N2—C16	−74.2 (6)	C12—C13—C14—C15	0.9 (12)
C5—N1—C1—C2	−0.7 (10)	C13—C14—C15—C16	0.0 (12)
Pd1—N1—C1—C2	172.3 (6)	C12—N2—C16—C15	0.5 (10)
N1—C1—C2—C3	0.1 (11)	Pd1—N2—C16—C15	176.3 (5)
C1—C2—C3—C4	1.5 (11)	C12—N2—C16—C17	−179.3 (7)
C2—C3—C4—C5	−2.6 (11)	Pd1—N2—C16—C17	−3.5 (10)
C1—N1—C5—C4	−0.3 (9)	C14—C15—C16—N2	−0.7 (11)
Pd1—N1—C5—C4	−172.6 (5)	C14—C15—C16—C17	179.1 (7)
C1—N1—C5—C6	178.5 (6)	N2—C16—C17—C22	−47.9 (10)
Pd1—N1—C5—C6	6.3 (9)	C15—C16—C17—C22	132.3 (8)
C3—C4—C5—N1	2.0 (11)	N2—C16—C17—C18	136.7 (7)
C3—C4—C5—C6	−176.9 (7)	C15—C16—C17—C18	−43.1 (10)
N1—C5—C6—C11	52.8 (9)	C22—C17—C18—C19	1.7 (11)
C4—C5—C6—C11	−128.4 (7)	C16—C17—C18—C19	177.3 (7)
N1—C5—C6—C7	−130.6 (7)	C17—C18—C19—C20	−2.0 (12)
C4—C5—C6—C7	48.2 (9)	C18—C19—C20—C21	1.1 (12)
C11—C6—C7—C8	−3.0 (10)	C19—C20—C21—C22	0.1 (12)
C5—C6—C7—C8	−179.7 (6)	C20—C21—C22—C17	−0.5 (12)
C6—C7—C8—C9	1.9 (11)	C18—C17—C22—C21	−0.5 (11)
C7—C8—C9—C10	−0.5 (11)	C16—C17—C22—C21	−175.9 (7)
